# Differential Changes in Postsynaptic Density Proteins in Postmortem Huntington's Disease and Parkinson's Disease Human Brains

**DOI:** 10.1155/2014/938530

**Published:** 2014-01-16

**Authors:** C. Fourie, E. Kim, H. Waldvogel, J. M. Wong, A. McGregor, R. L. M. Faull, J. M. Montgomery

**Affiliations:** ^1^Department of Physiology, Centre for Brain Research, University of Auckland, Private Bag 92019, Auckland, New Zealand; ^2^Department of Anatomy with Radiology, Centre for Brain Research, University of Auckland, Private Bag 92019, Auckland, New Zealand; ^3^School of Pharmacy, Centre for Brain Research, University of Auckland, Private Bag 92019, Auckland, New Zealand

## Abstract

NMDA and AMPA-type glutamate receptors and their bound membrane-associated guanylate kinases (MAGUKs) are critical for synapse development and plasticity. We hypothesised that these proteins may play a role in the changes in synapse function that occur in Huntington's disease (HD) and Parkinson's disease (PD). We performed immunohistochemical analysis of human postmortem brain tissue to examine changes in the expression of SAP97, PSD-95, GluA2 and GluN1 in human control, and HD- and PD-affected hippocampus and striatum. Significant increases in SAP97 and PSD-95 were observed in the HD and PD hippocampus, and PSD95 was downregulated in HD striatum. We observed a significant increase in GluN1 in the HD hippocampus and a decrease in GluA2 in HD and PD striatum. Parallel immunohistochemistry experiments in the YAC128 mouse model of HD showed no change in the expression levels of these synaptic proteins. Our human data show that major but different changes occur in glutamatergic proteins in HD versus PD human brains. Moreover, the changes in human HD brains differ from those occurring in the YAC128 HD mouse model, suggesting that unique changes occur at a subcellular level in the HD human hippocampus.

## 1. Introduction

Huntington's disease (HD) and Parkinson's disease (PD) are distinct neurodegenerative diseases that present with unique motor and cognitive symptoms. HD is an autosomal dominant inherited disease caused by the expansion of a polyglutamine repeat sequence in the huntingtin gene, which results in a progressive loss of medium spiny neurons in the striatum [1]. PD is a sporadic neurodegenerative disease, although there are rare familial cases. It is marked by the loss of dopaminergic neurons of the substantia nigra pars compacta, which leads to abnormal basal ganglia circuitry, resulting in motor symptoms [2, 3]. Treatments for these diseases are symptomatic and new therapeutic targets are of the essence. Emphasis is now being placed on the changes that occur at the synapse and the processes that underlie cognitive dysfunction as it has been shown that synaptic and cognitive dysfunction occurs long before the onset of clinical symptoms in the human [4–7].

Glutamate receptors are currently viewed as valuable therapeutic targets in both HD [6] and PD [7]. N-Methyl-D-Aspartate (NMDA) and alpha-amino-3-hydroxy-5-methyl-4-isoxazolepropionic acid (AMPA)-type glutamate receptors and their bound postsynaptic density-membrane associated guanylate kinases (PSD-MAGUKs) are critical for synapse development and plasticity [8–12]. MAGUKs act as scaffolding molecules and are responsible for maintaining the structure of synapses, trafficking of receptors, and activating signalling molecules. PSD-95 targets AMPA receptors to the synapse through its interaction with stargazin [13] and also binds directly to NMDA receptor subunits (GluN2A and GluN2B) for synaptic targeting [14, 15]. SAP97 binds directly to the GluA1 subunit of AMPA receptors to traffic them to the PSD [16] and the GluN2 subunits of NMDA receptors [17] and together with CASK traffic NMDA receptors through a unique secretory pathway to the PSD [18].

It is evident that MAGUKs could play a role in the pathogenesis of some neurodegenerative diseases [19, 20]. With regard to Huntington's Disease, normal huntingtin is associated with NMDARs via PSD-95 but mutant huntingtin impairs the interaction between PSD-95 and huntingtin, leading to excitotoxicity through increased NMDA receptor activity [21], which is a key feature of this neurodegenerative disease [22–24]. In the striatum of YAC128 HD model mice, increased levels of PSD-95 as well as increased PSD-95-GluN2B interactions are observed in extrasynaptic regions [25, 26]. A reorganisation of postsynaptic density proteins, including a switch of PSD-93 by PSD-95 in the striatum of the R6/1 HD mouse model, has also been described [27]. In the N171-82Q transgenic HD mouse model, a decrease in striatal PSD-95-like proteins was observed [28]. In Parkinson's Disease, there is a reduced interaction between NMDARs and MAGUKs in the striatum of 6-OHDA lesioned PD animal models [19], as well as a change in the subcellular distribution and levels of PSD-95 and SAP97 [29].

To date, animal models of HD and PD have provided valuable information on how synaptic structure and function may be altered in these human diseases; however the results with respect to changes in the glutamatergic synapse vary between studies and between models [20, 30]. To determine the changes that occur with neurodegenerative diseases of the human brain, it is imperative to examine the postmortem brain tissue of patients who died from these disorders. Here we have used postmortem human brain tissue to investigate whether changes in synaptic protein expression occur in response to human neurodegenerative disease and thereby may play an important role in the changes in synapse function that occur in disease. We focused on the MAGUKs PSD-95 and SAP97 as they play a major role in regulating glutamate receptor trafficking to synapses and also glutamate receptor localisation at synapses [8–10, 18, 31]. Our data show that changes occurring in PSD-95, SAP97 and glutamate receptor subunit expression in the human HD hippocampus differ from changes in the YAC128 HD animal model, suggesting that unique changes occur in the human brain in response to neurodegenerative disease that vary across different brain regions.

## 2. Materials and Methods

### 2.1. Human Brains

Human brain tissue was obtained from the Neurological Foundation of New Zealand Human Brain Bank (Centre for Brain Research, University of Auckland). The consent and research protocols used in this study were approved by the University of Auckland Human Participants Ethics Committee. The post-mortem human brain tissue was processed and dissected as described elsewhere [32, 33]. Briefly, brains were perfused via the basilar and carotid arteries with phosphate buffered saline (1% sodium nitrite) and then with 15% formalin in 0.1 M phosphate buffer (pH 7.4). Brains were dissected into the different functional parts including blocks of the striatum and hippocampus. The blocks were then cryoprotected in 20% sucrose 0.1 M phosphate buffer with 0.1% sodium-azide and frozen at −80°C. The hippocampal and striatal frozen tissue blocks were cut into 50 *μ*m coronal sections on a microtome and stored in PBS-azide at 4°C until used for immunohistochemistry. For hippocampal studies, a total of 34 human brains were examined, 12 control cases, 11 HD cases, and 11 PD cases (see Table 1). For the striatum, a total of 21 brains were examined, 7 control cases, 8 HD cases, and 6 PD cases (see Table 2). Numbers are presented as mean ± standard deviation.

### 2.2. Immunohistochemistry in Human Brain Sections

Immunohistochemistry was performed as previously described [32, 33]. Free-floating tissue sections were first incubated in PBS-Triton (0.2%) overnight at 4°C. Tissue was washed in citric acid buffer (pH 4.5) and sections then heated in the microwave for 30 seconds on high power for antigen retrieval if needed. The sections were cooled to room temperature and washed with PBS-T (3 × 10 min). The sections were then incubated in 50% methanol, 0.9% hydrogen peroxide solution for further antigen retrieval and for blocking endogenous peroxidise activity. Sections were incubated for 72 hours (4°C) in primary antibodies against mouse GluA2 (NeuroMab, 75-002) 1 : 500, rabbit PSD-95 (Sigma, HPA010122) 1 : 300, rabbit SAP97 (ABR, PA1-741) 1 : 1000, and mouse GluN1 (Millipore, MAB363) 1 : 300. All antibodies were checked for specificity by Western Blot analysis (see Figure  S1 in supplementary material available online at http://dx.doi.org/10.1155/2014/938530). Sections were then washed and incubated overnight at room temperature with the respective biotinylated secondary antibodies, goat anti-mouse (Sigma, B7264) 1 : 500 and goat anti-rabbit (Sigma, B7389) 1 : 1000. Sections were again washed and incubated in the tertiary antibody, extravadin peroxidase (Sigma, E2886) 1 : 1000 for 4 hours at room temperature. The chromogen was 0.05% 3,3-diaminobenzidine tetrahydrochloride (DAB; Sigma, D5637) and 0.01% H_2_O_2_ in 0.1 M phosphate buffer, pH 7.4 for 10–20 min. Sections were mounted, dehydrated, and cleared in xylene before being coverslipped and imaged. Omission of the primary antibody resulted in no immunoreactivity (not shown). Immunohistochemical staining was repeated a minimum of 3 times for each control, HD and PD cases.

### 2.3. YAC 128 Mice Immunohistochemistry

Twelve-month-old male and female YAC128 transgenic HD mice expressing the human huntingtin protein containing a 128 CAG repeat were utilised for this study. At this age the mice are highly symptomatic and comparable to late stage HD. YAC128 mice were purchased from Jackson Labs (FVB-Tg(YAC128)53Hay/J). Breeding pairs were established between male heterozygote and female wild-type animals. Mice were maintained on the FVB/N background strain and genotyped by PCR. Mice were housed and tissue harvested according to protocols approved by the University of Auckland Animal Ethics Committee. Animals were euthanized by rapid cervical dislocation and perfused with 4% paraformaldehyde. Coronal whole brain (30 *μ*m) free-floating tissue sections were first incubated in PBS-Triton (0.2%) overnight at 4°C. The sections were then incubated in 50% methanol, 0.9% hydrogen peroxide solution for further antigen retrieval and for blocking endogenous peroxidise activity. Hereafter the sections were incubated in 5% normal goat serum in PBST for 1 hour at room temperature. Sections were then incubated for 72 hours (4°C) in primary antibodies against mouse GluA2 (Alomone) 1 : 200 (WT *n* = 6, YAC *n* = 5), rabbit PSD-95 (Sigma) 1 : 200 (WT *n* = 6, YAC *n* = 5), rabbit SAP97 (ABR) 1 : 1000 (WT *n* = 6, YAC *n* = 5), and mouse GluN1 (Neuromab) 1 : 300 (WT *n* = 5, YAC *n* = 6). Procedures for secondary and tertiary antibody incubations were exactly as for the human brain sections, except that a 1 : 500 dilution was used. DAB procedures were for human brain sections.

### 2.4. Image J Densitometry

Densitometry analysis was performed using Image J (NIH USA, public domain). Densitometry analysis was performed for all MAGUK and glutamate receptor subunit immunostaining. Images were first collected on a Nikon TE2000 inverted microscope in bright-field mode. The imaging conditions were optimised for each antibody but kept constant for all control and diseased cases immunostained with each antibody. For each human case (control, HD, or PD), 10x z-stack images (1280 × 960 pixels) were taken in each hippocampal and striatal region at 2 *μ*m apart at 40x magnification, allowing the cell bodies and apical and basal dendrites to be clearly visible. For the hippocampus, images were collected in the dentate gyrus and CA3 and CA1 regions. In the striatum images were collected in the putamen and caudate nucleus. A minimum of 2 sections were collected from each hippocampal and striatal region in every human brain analysed. Z-stack images were converted to z-projections in Image J and the coloured images converted to grey scale images (8-bit). Background was measured on each z-projection image individually and automatically subtracted for each image. The image was then inverted so that density measurements were made in arbitrary units where a value of 255 is a complete transparency and a value of 0 is complete darkness. A similar method for analysis of DAB immunohistochemistry in the human brain using Image J has been used successfully [34]. Densitometric values for each immunohistochemistry set were normalised to the average of the normal cases immunostained in parallel to get the relative change in intensity compared to the control group (independent of experimental set). Intensity values for HD and PD tissues are presented as a ratio of the grey value divided by the control (non-diseased tissue) density value ± SEM. Quantitative immunohistochemical changes in hippocampal synaptic protein levels were also examined by quantitative Western Blot analysis to validate the consistency of the quantified changes (Figure S1). Statistical analysis was performed with SPSS (IBM Corporation 2010). Data are presented as mean ± SEM, and one way analysis of variance (ANOVA) or two-tailed Student's *t*-test was used to compare the densitometric measurements between control and diseased groups.

### 2.5. Western Blotting

Protein extracts from the human hippocampus of control and HD cases were denatured in Laemmli loading buffer (Sigma, S3401) at 95°C for 5 min. Protein extracts (30 *μ*g per sample) were separated by gel electrophoresis (NuPAGE 4–12% Bis-Tris gel; Invitrogen, NP0335) and transferred to polyvinylidene difluoride (PVDF) membrane (Amersham RPN303F). After blocking with 5% skim milk, the membranes were probed with primary antibodies and detected with species-specific horseradish-peroxidase-conjugated secondary antibodies (Millipore) and developed using ECL reagents (Amersham, RPN2132). Primary antibodies used were directed against GluR2 (Neuromab, 75-002) 1 : 500, PSD-95 (Sigma, HPA010122) 1 : 500, SAP97 (ABR, PA1-741) 1:500, and NR1 (Millipore, MAB363) 1 : 300. Ponceau S (Sigma) was used as a loading control [35]. The bands were visualised with the Fuji Film LAS-4000 scanner, and quantification of Western blot intensity was analysed using the Gel Analyser on Image J software (NIH USA, public domain) (Figure S1).

## 3. Results

### 3.1. Human Brain Western Blot and Immunohistochemistry Analysis Reveals Differential Changes in MAGUK Expression in the Diseased Human Brain

SAP97, PSD-95, GluA2, and GluN1 are known to play major roles at synapses within the dentate gyrus and areas CA3 and CA1 of the hippocampus [9, 10, 18, 31]. We were interested to determine (i) whether specific subregional changes occur in these proteins in HD or PD human hippocampus or striatum and (ii) whether the two neurodegenerative diseases PD and HD, which have different mechanisms of causation [1–3], differentially affect glutamatergic synaptic proteins in these brain regions. We therefore performed quantitative immunohistochemical and Western blot analysis of these glutamatergic synaptic proteins. The hippocampus was a major focus as many HD and PD patients have dementia, depression, cognitive decline, and other nonmotor symptoms as well as the classic well-characterised motor symptoms. Quantitative Western blot analysis of hippocampal tissue from control and HD post-mortem human brain tissue revealed that significant changes in synaptic protein levels were occurring with HD. Specifically, we observed that significant increases in the protein levels of PSD-95, SAP97, and the GluN1 subunit of the NMDA receptor occurred in HD hippocampal post-mortem tissue compared to controls (Figure S1), suggesting that differential changes are occurring in synaptic proteins in HD.

To provide more specific information on potential changes in immunoreactivity patterns of synaptic proteins in the principal neurons in HD and PD brain tissues compared to controls, we performed quantitative immunohistochemistry on human control HD and PD tissues. The expression of PSD-95, SAP97, and glutamate receptor subunits GluN1 and GluA2 were examined in the principle neurons of the dentate gyrus and CA3 and CA1 regions of the hippocampus, and in the caudate nucleus and putamen of the striatum. Immunohistochemistry and imaging criteria were kept constant for control and diseased cases to enable detection of changes in expression levels of the different synaptic proteins.

**Figure 1:**
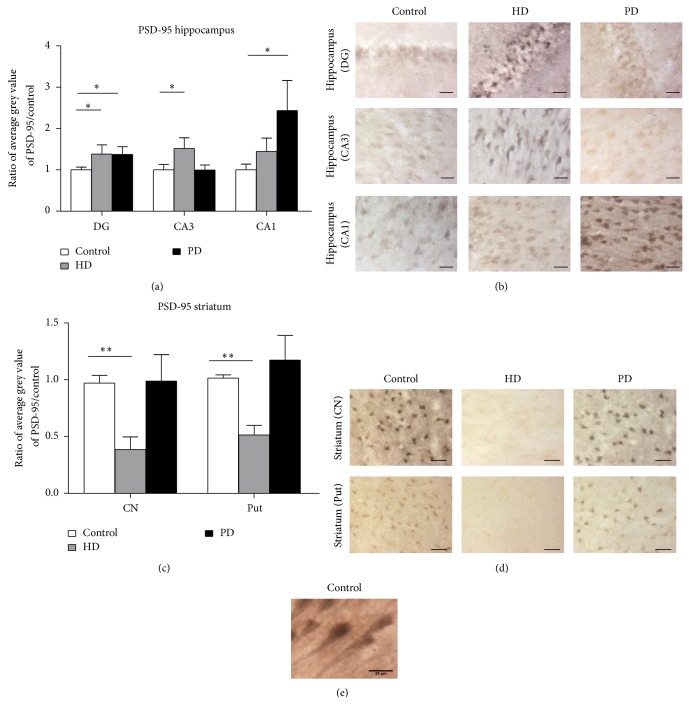
PSD-95 expression in the human HD and PD hippocampus and striatum. (a) PSD-95 significantly increased in DG and area CA3 in HD and in the DG and area CA1 in PD. ^*^ *P* ≤ 0.05. (b) PSD-95 significantly decreased in the HD striatum in both the CN (caudate nucleus) and Put (putamen). ^**^ *P* < 0.005. (c) Representative images of PSD-95 immunostaining in the dentate gyrus, CA3 and CA1 hippocampal regions. (d) Representative images of PSD-95 immunostaining in the caudate nucleus and putamen. Scale bar 25 *μ*m. (e) High power example image of PSD-95 immunolabeling showing the somatic and dendritic localisation patterns.

We first investigated expression changes in PSD-95 in the human hippocampus and striatum (Figures 1(a)–1(e)). Strong PSD-95 immunostaining was evident in the cell bodies and in the dendrites in both control and diseased hippocampal and striatal neurons (Figures 1(c)–1(e)). In HD postmortem brain, a significant 1.52 ± 0.26 fold increase in PSD-95 was observed in neurons in area CA3 of the hippocampus (*n* = 6, control *n* = 8; *P* < 0.05) and in the dentate gyrus (HD DG: 1.45 ± 0.22, *n* = 6, control *n* = 8, *P* = 0.05). No significant change was measured in neurons in area CA1 (HD CA1: 1.45 ± 0.31, *n* = 6, control *n* = 8, *P* = 0.13). In PD post-mortem tissue, a significant 1.37 ± 0.19 fold increase in the neurons in the dentate gyrus (PD *n* = 6, control *n* = 8; *P* < 0.05) and a significant 2.44 ± 0.73 fold increase in area CA1 (PD *n* = 6, control *n* = 8; *P* < 0.05; Figure 1(a)) regions were also observed. However, PSD-95 levels in the neurons in the CA3 region in PD were not significantly different from control levels (0.99 ± 0.12; PD *n* = 4, control *n* = 8).

In the striatum we observed stark changes in PSD-95 expression. In the HD striatum the expression of PSD-95 was significantly decreased in both the caudate nucleus (HD mean = 0.40 ± 0.12, *n* = 3, control *n* = 4; *P* < 0.005) and putamen (HD mean = 0.51 ± 0.09, *n* = 3, control *n* = 3; *P* < 0.005; Figure 1(b)). However, there were no changes in PD caudate nucleus (1.02 ± 0.25; PD *n* = 4, control *n* = 4) or putamen (1.16 ± 0.22; PD *n* = 4, control *n* = 4).

**Figure 2:**
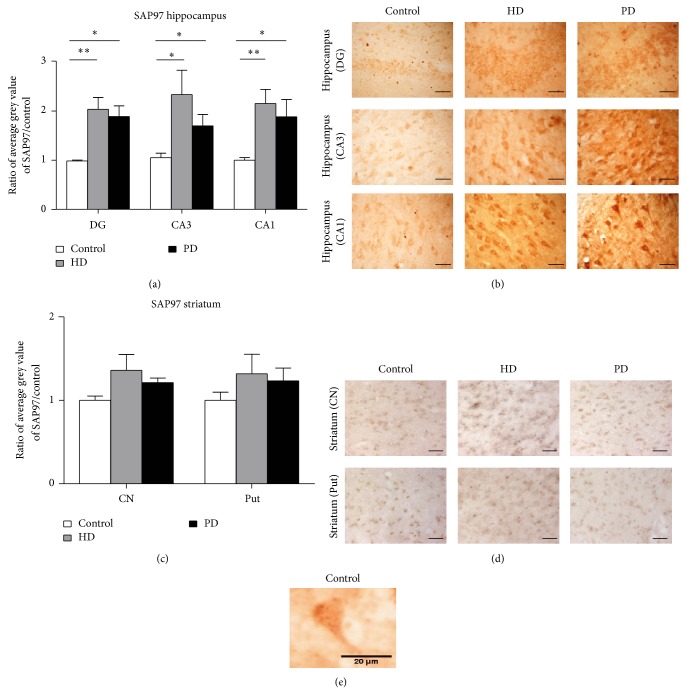
SAP97 expression in human HD and PD hippocampus and striatum. (a) Significant increases in SAP97 expression in the post-mortem human hippocampus in HD and PD patients. The significant increase in SAP97 expression occurs in all hippocampal regions examined: DG, area CA3, and area CA1. ^*^ *P* < 0.05, ^**^ *P* < 0.005. (b) No significant changes in SAP97 expression were observed in HD or PD striatum in either the caudate nucleus (CN) or the putamen (Put). (c) Representative images of SAP97 immunostaining in the dentate gyrus and CA3 and CA1 hippocampal regions. (d) Representative striatal images of SAP97 immunostaining in the putamen and caudate nucleus. Scale bar 25 *μ*m for (c) and (d). (e) High power example image of SAP97 immunolabeling, showing the expected somatic and dendritic localisations.

We next examined whether similar changes also occur in SAP97 expression and observed that the changes in SAP97 were different to those observed for PSD-95. Immunohistochemical analysis of hippocampal and striatal sections in dentate gyrus, CA3, CA1, and caudate nucleus, and putamen revealed that increases in SAP97 expression were found to occur in both the PD and HD human hippocampus but not in the striatum (Figures 2(a)–2(d)). SAP97 immunostaining was evident in the neuronal cell body layers and dendritic regions throughout the hippocampus and striatum (Figures 2(c)–2(e)). Consistent with its role in trafficking receptor complexes through the secretory pathway and along dendrites [18, 36], SAP97 appeared diffusely along dendrites (Figure 2(e)). SAP97 expression in each hippocampal region was found to increase to a similar degree in both HD and PD and occurred in both cell body and dendritic regions of the hippocampus. In the dentate gyrus, SAP97 was increased 2.06 ± 0.25 fold in HD (*n* = 6; *P* < 0.005) and similarly increased 1.91 ± 0.22 in PD (*n* = 9; *P* < 0.05) above control levels (*n* = 6). In the CA3 region, SAP97 was increased 2.22 ± 0.51 fold in HD (*n* = 5; *P* < 0.05) and 1.62 ± 0.26 fold in PD (*n* = 10; *P* < 0.05) above control (*n* = 6). In the CA1 region SAP97 was significantly increased 2.15 ± 0.31 fold in HD (*n* = 8; *P* < 0.005) and 1.88 ± 0.35 in PD (*n* = 6; *P* < 0.05) as compared to control (*n* = 6). In contrast, in the striatum there were no significant changes in the expression of SAP97 in the caudate nucleus in HD (*n* = 6) or PD (*n* = 5), nor in the putamen in HD (*n* = 6) or PD (*n* = 5) as compared to control (caudate nucleus *n* = 5, putamen *n* = 4; Figure 2(b)). Overall these results indicate that SAP97 expression is significantly altered throughout the hippocampus in human HD and PD, but not in the striatum. Moreover, these data show that the MAGUK proteins PSD-95 and SAP97 are differentially affected by different neurodegenerative diseases in the human brain and that these changes can differ in hippocampus versus striatum.

**Figure 3:**
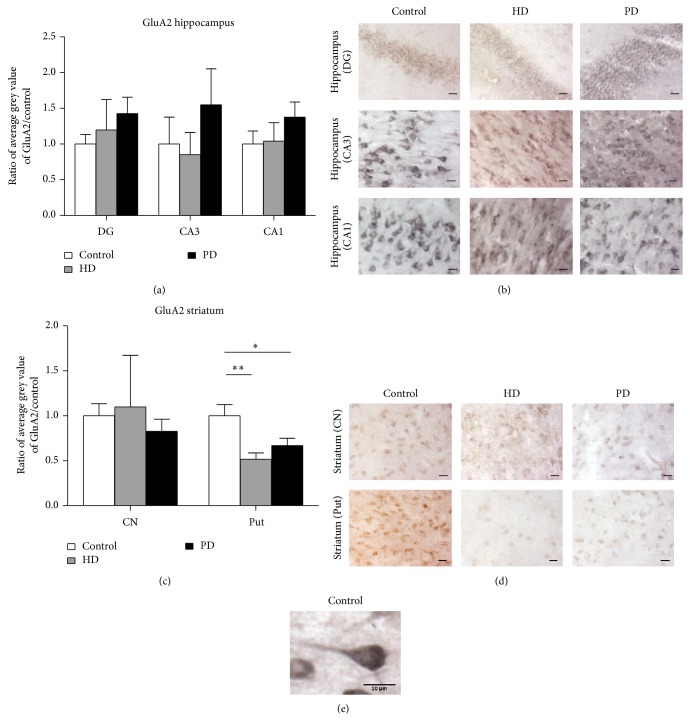
GluA2 expression levels in human postmortem HD and PD hippocampus and striatum. (a) No significant changes in GluA2 expression in HD or PD hippocampal regions. (b) GluA2 specific changes occur in the putamen (Put) of HD and PD tissue. ^*^ *P* < 0.05, ^**^ *P* < 0.005. (c) Representative images are shown for GluA2 in the dentate gyrus, CA3 and CA1 regions of the hippocampus. (d) Representative images of GluA2 immunostaining in the caudate nucleus and putamen of the striatum. Scale bar for (c) and (d) is 25 *μ*m. (e) High power example image of GluA2 immunolabelling, showing the somatic and dendritic localisations.

In the hippocampus, the majority of AMPA receptors are composed of GluA1/2 subunits and GluA2/3 subunits [37]. We performed quantitative immunohistochemistry to examine whether AMPA receptor subunit expression is altered in PD or HD in the human hippocampus and striatum by examining the expression of the GluA2 subunit common to both these receptor subtypes that could therefore reflect changes in either subtype of receptor (Figure 3). GluA2 immunostaining was again evident in the hippocampal and striatal neuronal cell bodies and the dendritic regions in both control and diseased human tissue (Figures 3(c)–3(e)). No significant changes in GluA2 subunit expression were observed in the HD or PD hippocampus dentate gyrus, area CA3, or area CA1, or in the striatal caudate nucleus (HD *n* = 4, PD *n* = 5, control *n* = 5; *P* > 0.05 in all cases; Figures 3(a)–3(d)). However, significant decreases in GluA2 expression were observed in the putamen relative to control levels (HD mean = 0.52 ± 0.07 of control levels, *n* = 4, *P* < 0.005; PD mean = 0.67 ± 0.08 of control, *n* = 5, *P* < 0.05; Figures 3(a)–3(d)).

**Figure 4:**
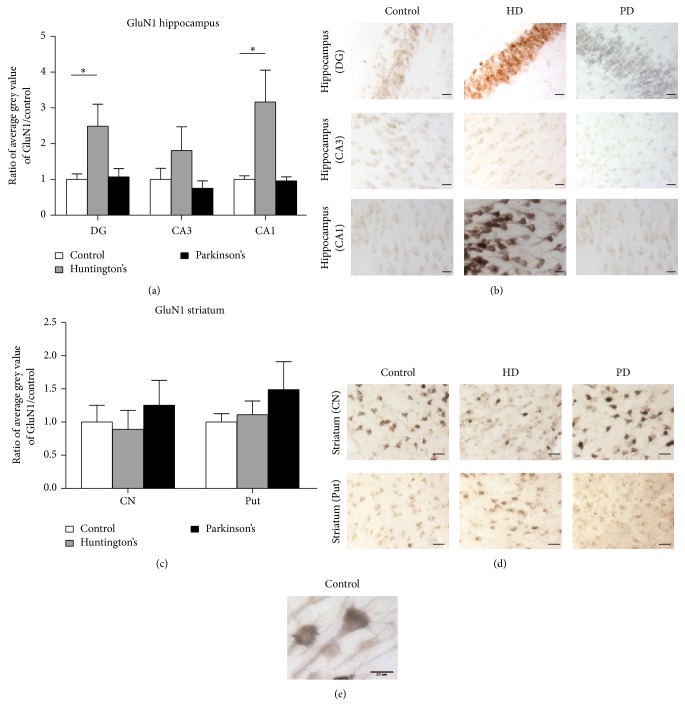
GluN1 expression levels in human postmortem HD and PD hippocampal and striatal tissues. (a) GluN1 significantly increased in the DG (dentate gyrus) and CA1 region in HD but no changes in GluN1 expression occur in PD hippocampus in any region. ^*^ *P* < 0.05. (b) No significant changes in GluN1 expression levels were observed in HD and PD striata in either the caudate nucleus (CN) or the putamen (Put). (c) Representative images are shown for GluN1 in the dentate gyrus and CA3 and CA1 regions of the hippocampus. (d) Representative striatal images of GluN1 immunostaining in the caudate nucleus and putamen. Scale bar 25 *μ*m for both (c) and (d). (e) High power example image of GluN1 immunolabelling, showing the somatic and dendritic localisations.

We also examined potential changes in the expression of GluN1, the obligatory subunit of the NMDA receptor (Figure 4). Strong GluN1 immunostaining was evident throughout the somatic and dendritic regions in both hippocampal and striatal neurons (Figures 4(c)–4(e)). We observed significant increases in GluN1 expression above control levels in neurons in the HD hippocampus (Figure 4(a)). Specifically, a significant 2.49 ± 0.62 fold increase occurred in the dentate gyrus (HD *n* = 8, control *n* = 8, *P* < 0.05) and a significant 3.17 ± 0.89 fold increase in area CA1 (HD *n* = 8, control *n* = 7, *P* < 0.05). No significant change was observed in neurons in area CA3 (*P* > 0.1). No changes occurred in PD hippocampus in either the dentate gyrus, area CA3, or area CA1 (PD *n* = 6, control *n* = 7). In the striatum, no change in GluN1 expression was observed in either disease in the caudate nucleus or putamen (HD *n* = 4, PD *n* = 4, control *n* = 4; Figure 4(b); *P* > 0.05 in all cases). Together these data show that different changes in NMDA and AMPA receptor subunit expression levels occur in the hippocampal and striatal human brain regions in response to HD versus PD.

### 3.2. Similar Changes in MAGUK Expression Do Not Occur in the Hippocampus of HD Animal Model YAC128

**Figure 5:**
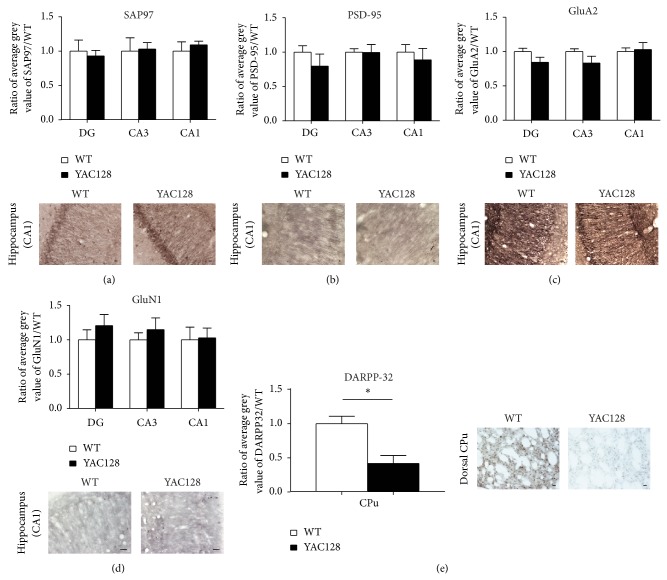
Quantitative immunohistochemistry of SAP97, PSD-95, GluN1, and GluA2 expression in YAC128 hippocampal sections. Sections were prepared from symptomatic 1-year-old YAC128 mice to provide a comparison to the end stage of human HD. (a)–(d). Top: Quantification of (a) SAP97, (b) PSD-95, (c) GluA2, and (d) GluN1 levels in dentate gyrus (DG), area CA3, and area CA1. Below: Example immunohistochemical staining for each glutamatergic synaptic protein in the hippocampal CA1 region in control (wildtype) and YAC128 mice. (e) Immunohistochemical quantification of DARPP-32 expression in wild-type and YAC128 striatum (caudate putamen, CPu). ^*^ *P* < 0.05.

We were particularly intrigued by the significant changes in the expression levels of PSD-95, SAP97, and GluN1 in the human HD hippocampus (Figures 1–4, Figure S1). Although HD is considered a motor disorder, there is significant evidence that cognitive effects appear before the motor symptoms suggesting a role of hippocampal changes in the disease [1, 38–40]. In animal models of HD, striatal changes in glutamatergic synapse structure and function have been widely addressed (e.g. [22, 24–26, 61]); however the hippocampus in YAC128 HD model mice has been shown to be spared of atrophy and degeneration [41, 42]. We wanted to determine whether the changes in MAGUK and glutamate receptor subunit expression we observed in the human hippocampus in HD were also occurring in the hippocampus of the YAC128 mouse model of HD. Immunohistochemistry was performed on hippocampal sections from YAC128 mice at 12 months of age, when the animals are highly symptomatic and in the late stage of HD and therefore are comparable to tissue from post-mortem human HD patients. As expected, SAP97, PSD-95, GluA2 and GluN1 immunostaining was observed strongly in the dendritic regions of neurons in area CA1, CA3, and dentate gyrus (Figures 5(a)–5(d)). However, densitometry quantification revealed that none of the changes in SAP97, PSD-95, and GluN1 that we observed in human post-mortem HD hippocampus occurred in YAC128 mice hippocampus. We observed no significant changes in expression levels for any of these synaptic proteins compared to control tissue in either dentate gyrus, area CA1, or area CA3 of YAC128 hippocampus (Figure 5). To ensure that our immunohistochemical analysis could detect changes in protein expression levels in YAC128 tissue, we examined the expression changes of DARPP-32 to act as a positive control in both YAC128 and wildtype striatum. DARPP-32 is a marker of dopaminergic neurons in the striatum and has been reported to decrease in YAC128 striatum [41, 43–45]. Indeed we observed that DARPP-32 levels were significantly decreased in YAC128 striatum to 0.42 ± 0.12 of wildtype control levels (*n* = 5; *P* < 0.05; Figure 5). Therefore our data suggest that different subcellular hippocampal changes are occurring in human HD compared to animal models of the same disease.

## 4. Discussion

Here we report changes that occur in SAP97, PSD-95, GluN1 and GluA2 in the human brain in response to the neurodegenerative diseases HD and PD. Our data reveal that the overall patterns of change in these synaptic proteins are not the same in the post-mortem human HD versus PD brains, with the majority of the changes observed occurring in HD brains. We also report that the changes observed differed between the hippocampus versus striatum, as evidenced by increases in glutamatergic synaptic proteins in the hippocampus but only decreases in the striatum. Moreover, hippocampal increases tended to occur across all hippocampal regions examined (dentate gyrus, CA3, and CA1), whereas some putamen-specific changes were observed in the striatum. We predict that the hippocampal-specific increases in SAP97, PSD-95 and GluN1 are likely related to the nonmotor symptoms of HD such as cognitive decline or dementia [38, 46, 47], while the striatal-specific decreases in PSD-95 and GluA2 are related to striatal degeneration and behavioural motor symptoms. As the hippocampal changes did not occur in the YAC128 HD mouse model, our data suggest that unique changes occur in the human hippocampus with HD.

### 4.1. Major Changes in PSD-95 and SAP97 Occur in HD and PD Human Brain

One of the major increases that we observed occurred in SAP97 in the hippocampus of HD and PD brains. This suggests a hippocampal-specific role of SAP97 in HD and PD as no changes in SAP97 expression levels occurred in the striatum despite other significant subcellular pathology [1, 2]. Therefore SAP97 expression changes do not appear to be involved in the subcellular striatal changes that underlie the motor symptoms of PD and HD. It remains to be determined whether the specific SAP97 hippocampal changes are a pathological hallmark of the cognitive changes seen in HD and PD patients [4, 38, 46, 47]. At the cellular level, the known role of SAP97 in regulating AMPA and NMDA receptor-mediated postsynaptic currents [9, 10, 31] and NMDA receptor-dependent excitotoxicity [48], suggests that the increases in SAP97 alter the trafficking, synaptic expression and/or localisation of NMDA and AMPA receptors in the diseased brain.

Changes in PSD-95 also occurred in the hippocampus in HD and PD post-mortem tissue, and the lack of change in striatal PD tissue suggests it also does not play a role in the striatal pathology of PD. However, subcellular decreases in PSD-95 levels do appear to play a major role in the subcellular striatal pathology of HD. This is in agreement with Western Blot analysis of human HD brain [27]. As PSD-95 is a major component of the postsynaptic density at excitatory synapses, these data suggest that the decrease in PSD-95 observed could be secondary to a loss of synapses. This is consistent with previous work in human HD tissue in which Golgi impregnation has shown alterations in the number and size of dendritic spines of striatal medium spiny neurons [49]. Similar spine pathology has also been observed in transgenic R6 HD mice [50, 51]. However, no similar decrease was observed for SAP97 which is also a major component of the PSD, suggesting that changes beyond synapse number may also underlie the decrease in PSD-95. The consequence of this decrease is not known, but it may be an attempt to reduce NMDA receptor-mediated excitotoxicity [9, 48]. In addition, given the importance of PSD-95 in the synaptic targeting of AMPARs [13], the reduced expression of PSD-95 in the HD striatum may also relate to the observed HD striatal reduction in GluA2.

With regards to changes in glutamate receptor subunit expression, our data show that GluN1 and GluA2 expression levels are differentially affected by hippocampal versus striatal subcellular pathology that occurs in PD or HD human brain. For example, GluN1 was not altered in the human HD striatum, despite early work showing that NMDA receptor binding was significantly decreased the putamen in human HD tissue [52]. GluN1 was significantly increased in the human HD hippocampus, which may again reflect a role in cognitive changes in HD. In contrast, no changes in GluN1 were observed in PD post-mortem brain. However, the precise sub-synaptic location of the expression of the obligatory GluN1 subunit will be important to determine in both the HD and PD human striatum and hippocampus, and this may explain the differential changes observed. In the YAC128 animal model of HD, extrasynaptic NMDA receptors in the striatum are upregulated and their blockade reverses the HD-induced signalling and motor learning deficits [53–55]. Whether the observed upregulation of GluN1 in the human hippocampus represents an increase in extrasynaptic receptors is not yet known. Our observed lack of change in GluN1 in the YAC128 hippocampus suggests the change in NMDA receptor distribution in HD may be restricted to the striatum, or alternatively that total NMDA receptors level remain the same but are simply redistributed to extrasynaptic regions. Interestingly, NMDA receptor localisation is differentially regulated by *α*- and *β*-isoforms of SAP97 [10]. It will be of significant interest to determine whether the upregulation of SAP97 observed in human HD and PD brain is specific to *α*- or *β*SAP97 isoforms that regulate synaptic versus extrasynaptic NMDA receptors. In contrast to GluN1, the decrease in GluA2-containing AMPARs was restricted to the putamen in both HD and PD post-mortem brain tissue suggesting that changes in AMPAR subunits do not contribute to hippocampal changes in HD and PD. However, a decrease in GluA2-containing receptors in the putamen in PD and HD could alter AMPAR-mediated synaptic transmission in the basal ganglia and consequently play a more dominant role in the motor symptoms of PD and HD.

Currently, it is unknown whether the observed increases in hippocampal MAGUK and GluR subunit expression levels helps or hinders neuronal survival, synaptic transmission, synaptic plasticity, or cognition and, whether the increases represent the active decline of neuronal structure and function or represent proactive changes in an attempt to restore lost synapse function. Moreover, these increases may differ depending on the HD grade, CAG repeat length, or sex of the patient. The limited availability of human tissue makes these correlations difficult to obtain. It is also not yet known if the observed changes in synaptic proteins occur at synapses. Unfortunately, the use of human tissue precludes quantification of proteins at synaptic sites via double immunolabelling due to the lipofuscin-induced autofluorescence that interferes with synaptic staining [56]. Reduced synaptic plasticity is routinely seen in Huntington's disease mouse models [57–59], which are proposed to underlie the observed cognitive changes. Whether similar changes in synaptic plasticity occur in the human HD brain has so far not been able to be determined but remains of significant interest.

### 4.2. Comparative Changes in Synaptic Protein Expression in Human versus Animal Tissue

The major changes in glutamatergic synaptic protein expression that we observed in the HD human hippocampus were not found to occur in the YAC128 animal model of HD. In addition, we observed that the staining pattern of PSD-95, SAP97, GluN1 and GluA2 were not identical in human and animal tissue. Our observed diffuse dendritic expression of these synaptic proteins in human tissue is consistent with previous work in the human brain revealing the mostly diffuse expression of PSD-95 [60]. Whether this expression pattern reflects a higher proportion of synaptic protein trafficking along dendrites in the human tissue remains to be determined.

The changes in synaptic protein expression that we observed in the human HD tissue may reflect changes that only occur at end-stage HD. Our YAC128 mice were highly symptomatic, late-stage HD at the time of our experiments. Therefore future studies are required to examine whether any changes in synaptic proteins occur later when the YAC128 mice are end stage (at ~18 months in our colony). It will also be important to determine whether these human versus mice differences are consistent in other HD animal models. Previous work on YAC128 mice has largely focused on striatal synaptic changes [22–26, 55, 61], and the lack of hippocampal changes we observed likely reflects the previous observation that the hippocampus in YAC128 (but not R6/2) HD model mice are spared of atrophy and degeneration [41, 42, 62]. This suggests that the previously described cognitive dysfunction, depressive behaviour, increased mutant huntingtin nuclear localization, and decreased hippocampal neurogenesis observed in YAC128 mice [41, 63–65] are independent of changes in the expression of the synaptic proteins examined in the present study. Previous work has described increased PSD-95 localisation and PSD-95-GluN2B interactions in striatal extrasynaptic fractions [12]. Together with previous work showing an increase in extrasynaptic NMDA receptors [55], these data suggest that a redistribution of PSD proteins occurs in YAC128 animals. Therefore despite a lack of change in synaptic protein levels detected in our study in YAC128 animals, a change in their subsynaptic distribution or function could underlie YAC128 subcellular pathology. However, our immunohistochemical analysis does reveal that synaptic proteins are differentially altered in the HD human versus mouse brain and that unique changes may occur in the human hippocampus with HD making further study of human tissue of significant importance.

Inconsistencies in reported changes in protein expression are not restricted to human versus animal data but also occur between different animal models of the same disease. We observed no change in PSD-95 expression in the hippocampus of YAC128 mice; however in contrast in HD R6/1 mice a significant decrease in PSD-95 levels has been observed [66]. Other studies examining changes in synaptic proteins in animal models of HD and PD have also shown differential changes in PSD-95 as well as GluN1 [19, 24, 27–29, 67–69]. The reasons underlying these conflicting data likely result not only from the mouse model employed but also from the symptomatic stage and quantitative methodology used. We believe that it is imperative to assess which of the changes described in animal models are directly reflective of the changes occurring in the human brain.

However, unlike human cellular studies, animal models enable examination of neuronal changes occurring in the pre-symptomatic and symptomatic phases of the disease. Human tissue from presymptomatic and symptomatic phases is understandably extremely rare. The changes that occur at these stages will be important to determine, but currently cellular human brain studies are largely restricted to end stage studies. Inherent variability in the measurements of changes in protein expression is also more evident in data from human brain tissue. This is not occurring in animal studies and is likely due to the use of genetically similar laboratory animals, enabling changes to be more easily deciphered. However, despite the inter-patient variation, our observed changes in SAP97, PSD-95, GluA2, and GluN1 were routinely observed across all patients within a disease group, reflecting consistent changes across the spectrum of patients.

## 5. Conclusions

The overall changes in SAP97, PSD-95, GluA2, and GluN1 levels in HD versus PD postmortem human brains represent unique disease-related changes occurring differentially in discrete brain regions. These changes likely reflect the different origins, symptoms, and potentially different neurodegenerative mechanisms of these two diseases. We hypothesise that the hippocampal increases in SAP97, PSD-95, and GluN1 may be a pathological hallmark of the cognitive changes seen in HD patients [4, 38, 46]. Overall, a lack of human data has made it difficult to predict therapeutic outcomes in the human and to extrapolate animal model data to the human. However, here we have shown that unique changes in synaptic protein expression occur in the human hippocampus and striatum which establish a baseline comparison for animal models and should be considered in future studies.

## Supplementary Material

Supplementary Figure 1: Top: Antibody specificity as determined by Western Blot analysis of PSD-95, SAP97, GluA2 and GluN1 in human hippocampus. Example Western Blots are shown for PSD-95 (showing a doublet at ~82kDa band), SAP97 (~140 kDa band), GluA2 (~98kDa band) and GluN1 (~110kDa band) [18,31,70-73] in human control hippocampus. Bottom: Quantitative Western Blot analysis of PSD-95, SAP97, GluA2 and GluN1 in human control (black bars) and HD (gray bars) hippocampus show similar changes to quantitation by immunohistochemistry. ∗p < 0.05. Example protein bands are shown for PSD-95 (showing a doublet at ~82kDa band), SAP97 (~140 kDa band), GluA2 (~98kDa band) and GluN1 (~110kDa band) in human control (left) and HD (right) hippocampus.

## Figures and Tables

**Table 1:** Summarised details of cases used for the hippocampus immunohistochemistry.

Group	Number of cases	Age (years)	Sex	PM delay (hrs)	Pathology
Control	12	67.1 ± 15.7	9 males 3 females	17.3 ± 4.7	Normal

HD	11	65.9 ± 9.8 Onset age: 47 ± 8.6	9 males 2 females	12.7 ± 4.3	Grade 1–4. Average CAG repeat length: 43.64 ± 2.50

PD	11	79.6 ± 6.1	8 males 3 females	16.0 ± 9.3	Parkinson's Disease

**Table 2:** Summarised details of cases used for the striatum immunohistochemistry.

Group	Number of cases	Age (years)	Sex	PM delay (hrs)	Pathology
Control	7	74.0 ± 6.8	5 males 2 females	20.2 ± 3.2	Normal

HD	8	67.0 ± 12.0 Onset age: 48.5 ± 12.6	6 males 2 females	14.5 ± 6.9	Grade 1–4 Average CAG repeat length: 43.14 ± 3.44

PD	6	80.0 ± 5.9	4 males 2 females	11.1 ± 5.4	Parkinson's Disease
